# Factors affecting viability of *Bifidobacterium bifidum* during spray drying

**DOI:** 10.1186/s40199-014-0088-z

**Published:** 2015-01-25

**Authors:** Zahra Shokri, Mohammad Reza Fazeli, Mehdi Ardjmand, Seyyed Mohammad Mousavi, Kambiz Gilani

**Affiliations:** Department of Chemical Engineering, Islamic Azad University-Tehran South Branch, Tehran, Iran; Probiotic Research Laboratory, Department of Drug and Food Control, Pharmaceutical Sciences Research Center, Faculty of Pharmacy, Tehran University of Medical Sciences, Tehran, Iran; Biotechnology Group, Chemical Engineering Department, Tarbiat Modares University, Tehran, Iran; Aerosol Research Laboratory, Department of Pharmaceutics, Faculty of Pharmacy, Tehran University of Medical Sciences, Tehran, Iran

**Keywords:** Spray drying, *Bifidobacterium bifidum*, Viability, Moisture, Response surface methodology

## Abstract

**Background:**

There is substantial clinical data supporting the role of *Bifidobacterium bifidum* in human health particularly in benefiting the immune system and suppressing intestinal infections. Compared to the traditional lyophilization, spray-drying is an economical process for preparing large quantities of viable microorganisms. The technique offers high production rates and low operating costs but is not usually used for drying of substances prone to high temperature. The aim of this study was to establish the optimized environmental factors in spray drying of cultured bifidobacteria to obtain a viable and stable powder.

**Methods:**

The experiments were designed to test variables such as inlet air temperature, air pressure and also maltodextrin content. The combined effect of these variables on survival rateand moisture content of bacterial powder was studied using a central composite design (CCD). Sub-lethal heat-adaptation of a *B. bifidum* strain which was previously adapted to acid-bile-NaCl led to much more resistance to high outlet temperature during spray drying. The resistant *B. bifidum* was supplemented with cost friendly permeate, sucrose, yeast extract and different amount of maltodextrin before it was fed into a Buchi B-191 mini spray-dryer.

**Results:**

Second-order polynomials were established to identify the relationship between the responses andthe three variables. Results of verification experiments and predicted values from fitted correlations were in close agreement at 95% confidence interval. The optimal values of the variables for maximum survival and minimum moisture content of *B. bifidum* powder were as follows: inlet air temperature of 111.15°C, air pressure of 4.5 bar and maltodextrin concentration of 6%. Under optimum conditions, the maximum survival of 28.38% was achieved while moisture was maintained at 4.05%.

**Conclusion:**

Viable and cost effective spray drying of *Bifidobacterium bifidum* could be achieved by cultivating heat and acid adapted strain into the culture media containing nutritional protective agents.

## Introduction

Probiotics are live microbial feed supplements that beneficially affect hosts by improving its intestinal microbial balance [1]. Bacterial strains selected as probiotics are predominantly from the genera *Bifidobacteria* and *Lactobacilli,* which are indigenous to the human gastrointestinal tract [2]. These strains possess unique ability to establish in the human intestine and are associated with restoration of normal intestinal flora by outcompeting harmful flora and human pathogens [3]. They are also believed to have detoxifying ability against mycotoxins [4]. Because of their positive effect on host’s health, production and consumption of live probiotic supplements and food products enriched with friendly microorganisms have been of focus [5]. Both freeze-dying and spray-drying which are currently used to dry probiotic cultures expose the culture to extreme environmental conditions [6]. Spray drying is however more economic and efficient because of its continuous high production rate behavior, but viability of bacteria is usually affected due to use of extreme heat [7].

During spray drying bacteria are exposed to multiple stresses, i.e. heat (both wet and dry), oxidation, dehydration-related stresses (osmotic, acidic and thermal shock, accumulation of toxic compounds, etc.) which potentially could lead to cell death. Loss of viability appears to be principally caused by cell membrane damage [8]; moreover, the cell wall, ribosome and DNA are also affected at higher temperatures [9].

Thermal shock is the most influential factor in this field. Compared to the untreated bacteria, those which are pre-treated in water bath are usually more resistant to dry heat of outlet air temperature during spray drying [10]. High temperatures could lead to heat or stress proteins. The induction of heat shock on bacterial has led to the production of heat shock protein (HSP) or stress proteins. The role of protective proteins is to prevent malicious connections between intracellular amino acids. These proteins are produced by the genes present in all living cells. In 2005, Joana Silva and colleagues showed that the growth of the bacteria in non-controlled pH conditions results in induction of heat shock proteins and results in more bacteria to survive during spray drying and storage [11]. Also water drainage which contributes to the stability of biological molecules and probiotic strains, may cause irreversible changes in the structural and functional integrity of bacterial membranes and proteins. Preservation of these essential functions and structure is crucial for the survival of bacteria and the retention of their functionality.

The residual moisture content should be low enough to prevent damage to the product during storage. Too low moisture content of probiotic powders can also be injurious [8]. Humidity below 2% is also harmful because it can increase the risk of oxidation of unsaturated fatty acids in the cell membrane of bacteria and it can destroy the units of hydration around these fatty acids [12]. Based on the measurements of glass transition temperature (*T*_*g*_), critical water content 4-7% (w/v) is necessary and appropriate for the storage of culture powders at room temperature of 25°C [13,14].

As data on optimized spray drying of *B. bifidum* is trace we have tried to investigate the optimum spray drying conditions for preparation of viable *B. bifidum* powder with suitable moisture content.

## Materials and methods

### Microorganism and cultivation conditions

The bacterial strain of *Bifidobacterium bifidum PTCC 1644* (Persian Type Culture Collection- Iran) was previously adapted to gastrointestinal conditions such as acid, bile and NaCl [15].

### Heat adaptation of bacterial cultures

Bacteria underwent heat adaptation according to Jewell and Kashket [16]. Test tubes containing aliquots of 20 ml of 30 hours fresh bacterial culture (37°C and 5% CO_2_) in MRS broth (Merck GmbH, Germany) were treated at 60°C for 15 minutes. The survived and heat adapted strains were collected after further incubation of viable strains on MRS agar medium and after 48 hours incubation (temperature, 37°C and 5% CO_2_). The experiments were repeated at higher temperatures of 65°C and 75°C and the adapted strains were stored at -80°C for subsequent use in the spray drying. Strains subcultured on MRS broth were enriched with 0.05% L-cysteine (Merck GmbH, Germany), at 37°C for 30 hours [15]. Following incubation under 5% CO_2_ cells were harvested by centrifugation at 2000 rpm for 15 min, and were further re-suspended in sterile PBS-glycerol (20% v/v) solution and finally stored in 1mlcryotubes at -80°C.

### Preparation of spray drying feed suspensions

All feed solutions contained 10% permeate powder (Shirpooyan Yazd Co., Iran), 2.5% saccharose, 2.5% yeast extract as well as 2-6% maltodextrin (Merck GmbH, Germany) and were autoclaved at 121°C for 15 min before use.

A cryo-tube containing 1ml of the adapted *Bifidobacterium bifidum*was inoculated into the feed and was further incubated anaerobically (H2/CO2/N2; 10:5:85, Anoxomat WS8000, Mart_ Microbiology, Lichtenvoorde, Netherlands) at 37°C for 30 hours. The harvested feed contained 10^8^-10^9^cfu/ml prior to spray drying.

### Spray drying condition

A mini spray-dryer Buchi B-191 (Buchi, Flawil, Switzerland) and the adopted protocol of Johnson and Etzel [17] was used. The feed solution was transformed from a fluid state into a dried form by spraying it into a hot drying air. The process involved atomization of a liquid feedstock into a spray of droplets. Independent variables for optimized method of spray drying process design included:

- atomizing air pressure (bar)
- inlet air temperature (°C)
- outlet air temperature (°C)
- flow rate of fees suspension ($\frac{ml}{min}$)
- flow rate of drying air (aspiration ($\frac{m^3}{h}$))

The aspiration was set on 80% in all runs. The outlet temperature measured between drying chamber and cyclone was regarded as the drying temperature. Adjustment of outlet temperature was performed by holding flow rate of the feed suspension at a constant value (25% pump capacity ~ 5 ml min^−1^) for all outlet temperatures. The inlet temperature was varied, as shown in Table 1.

**Table 1:** **The level of variables in central composite design (CCD)**

**Factor**	**Low axial** **(-** ***α*** ** = − 1.68** **)**	**Low factorial** **(-1)**	**Center** **(0)**	**High factorial** **(+1)**	**High axial** **(+** ***α*** ** = + 1.68** **)**
A: Inlet temperature (°C)	79.77	90	105	120	130.23
B: Air pressure (bar)	3.32	4	5	6	6.68
C: Maltodextrin $\left(\frac{gr}{ml}\right)$	0.64	2	4	6	7.36

### Design of experiments and statistical modeling

Response surface methodology is a combination of mathematical and statistical techniques used for developing, improving and optimizing the processes. It is used to evaluate the relative significance of several affecting factors, even in the presence of complex interactions [18,19]. The most popular response surface methodology is the central composite design (CCD) [20], which was used to design the experiment. CCD has three set of experimental runs: (1) fractional factorial runs in which factors are studied at +1 and -1 levels; (2) center points that all factors are at their center levels, which aids with determining the curvature and replication, helps to estimate pure error; and (3) axial points, which are similar to center point, but one factor takes the values above and below the median of the two factorial levels, typically both outside their range. Axial points make the design rotatable [21]. Empirical models describing the experimental results were developed using data collected from the designed experiments and were generated using the least-squares method. Model parameters were estimated using a second-order model of the form (Eq. (1)) [22]:

1 $$ Y={\beta}_0+{\displaystyle \sum_{i=1}^k}{\beta}_i{X}_i+{\displaystyle \sum_{i=1}^k}{\displaystyle \sum_{j=1}^k}{\beta}_{ij}{X}_i{X}_j $$

Where *Y* is the expected value of the response variables, *β*_0_, *β*_*i*_, *β*_*j*_ are the model parameters, *X*_*i*_ and *X*_*j*_ are the coded factors evaluated, and *k* is the number of factors being studied. In this study, inlet air temperature, air pressure and maltodextrin concentration were selected as main factors. As shown in Table 1, each factor was examined in five levels, whereas the other parameters were kept constant. Accordingly, 20 experiments were conducted with 14 experiments organized in a factorial design and the 6 remaining experiments were involved in the replication of the central point to get good estimate of experimental error. The statistical software package, Design-Expert 7.0.0 (Stat-Ease, Inc., Minneapolis, MN, USA), was used for both the regression analysis of the experimental data, and the plot of the response contours and surface graphs. DX–7 is the windows-compatible software which provides efficient design of experiments (DOEs) for identification of vital factors that affect the process and uses RSM to determine optimal conditions [23,24]. The optimization module in DX–7 searches for a combination of factor levels that simultaneously satisfy the requirements placed on each of several responses [25,26].

### Enumeration of *Bifidobacterium bifidum*

Colony forming units (CFU) of the individual runs of bifidobacterial cultures before and after spray drying were determined by serial dilution of feed suspension and powders, followed by pour plating into MRS agar. Plates were incubated at 37°C, for 48 hours, under anaerobic condition. Survival rates were calculated as follows: Survival (%) = *N*/*N*0 × 100, where *N*0 and *N* represent the number of bacteria before and after drying respectively.

### Determination of moisture content in spray dried powders

Moisture content of spray dried powder which is defined as the ratio of dried water to initial powder weight, was determined by oven-drying at 102° [27]. This involved determination of the difference in weight before and after oven-drying. Moisture content was then expressed as a percentage of initial powder weight.

## Results and discussion

Twenty experiments were designed using CCD. The design matrix and the corresponding results of CCD experiments to determine the effects of the three independent variables are shown in Table 2.

**Table 2:** **Experimental plan and results of spray drying of** ***B. bifidum***

**Run**	**Factors**			**Responses**	
	**A (** **°C** **)**	**B (bar)**	**C (** $\frac{\mathbf{gr}}{\mathbf{ml}}$ **)**	**S (** **%** **)**	**Moisture (** **%** **)**
1	105.00	5.00	4.00	29.80	4.40
2	90.00	4.00	6.00	30.25	6.19
3	105.00	5.00	4.00	28.52	4.10
4	120.00	4.00	2.00	24.80	4.10
5	130.23	5.00	4.00	3.12	2.98
6	105.00	3.32	4.00	35.30	5.34
7	105.00	5.00	4.00	28.30	4.46
8	120.00	6.00	6.00	9.72	3.30
9	120.00	6.00	2.00	6.83	4.29
10	105.00	5.00	0.64	36.78	6.37
11	105.00	5.00	7.36	41.18	4.56
12	105.00	5.00	4.00	28.40	4.49
13	79.77	5.00	4.00	33.90	7.19
14	90.00	6.00	6.00	30.06	6.04
15	120.00	4.00	6.00	17.91	3.66
16	105.00	6.68	4.00	25.50	4.83
17	105.00	5.00	4.00	28.35	4.40
18	90.00	6.00	2.00	38.78	5.89
19	105.00	5,00	4,00	28,50	4.40
20	90.00	4.00	2.00	33.33	5.15

Quadratic model was found to be adequate for the prediction of the response variables.

2 $$ {Y}_1=+28.82-9.15\mathrm{A}-2.74\mathrm{B}-0.62\mathrm{C}-3.93\mathrm{AB}+0.98\mathrm{AC}+0.52\mathrm{B}\mathrm{C}-4.72{\mathrm{A}}^2-0.51{\mathrm{B}}^2+2.52{\mathrm{C}}^2 $$

3 $$ {Y}_2=+4.39-1.10\mathrm{A}-0.032\mathrm{B}-0.24\mathrm{C}-0.095\mathrm{AB}-0.33\mathrm{AC}-0.18\mathrm{B}\mathrm{C}+0.16{\mathrm{A}}^2+0.16{\mathrm{B}}^2+0.29{\mathrm{C}}^2 $$

Where *Y*_1_ and *Y*_2_, predicted Survival rate (%) and Moisture content (%) respectively; A is Inlet air temperature level; B is air pressure level; and C is maltodextrin concentration level. The statistical significance of the model equations (Eqs. (2)–(3)) and the model terms were evaluated by the F-test for analysis of variance (ANOVA), which indicated that the regressions were statistically significant. The results of analysis of variance (ANOVA) of the developed models are shown in Table 3. It illustrates that the two fitted models are significant with 95% confidence intervals (p-value < 0.05).

**Table 3:** **Analysis of variance for response surface models**

**Responses**		**Sum of square**	**DOF**	**Mean square**	**F-value**	**P-value**
	Model	1835.55	9	203.95	12.77	0.0002
	A-temperature	1142.75	1	1142.75	71.56	<0.0001
	B-pressure	102.32	1	102.32	6.41	0.0298
	C-maltodextrin	5.17	1	5.17	0.32	0.5820
	AB	123.40	1	123.40	7.73	0.0195
**Survival (** **%** **)**	AC	7.61	1	7.61	0.48	0.5058
	BC	2.14	1	2.14	0.13	0.7218
	A^2^	320.76	1	320.76	20.09	0.0012
	B^2^	3.81	1	3.81	0.24	0.6359
	C^2^	91.48	1	91.48	5.73	0.0377
	Residual	159.69	10	15.97		
	Model	20.16	9	2.24	11.57	0.0003
	A-temperature	16.48	1	16.48	85.06	<0.0001
	B-pressure	0.014	1	0.014	0.072	0.7933
	C-maltodextrin	0.79	1	0.79	4.08	0.0711
	AB	0.072	1	0.072	0.37	0.5551
**Moisture (** **%** **)**	AC	0.86	1	0.86	4.43	0.0616
	BC	0.26	1	0.26	1.34	0.2742
	A^2^	0.37	1	0.37	1.89	0.1996
	B^2^	0.37	1	0.37	1.89	0.1996
	C^2^	0.076	1	0.076	2.12	0.1765
	Residual	1.94	10	0.19		

**Figure 1:**
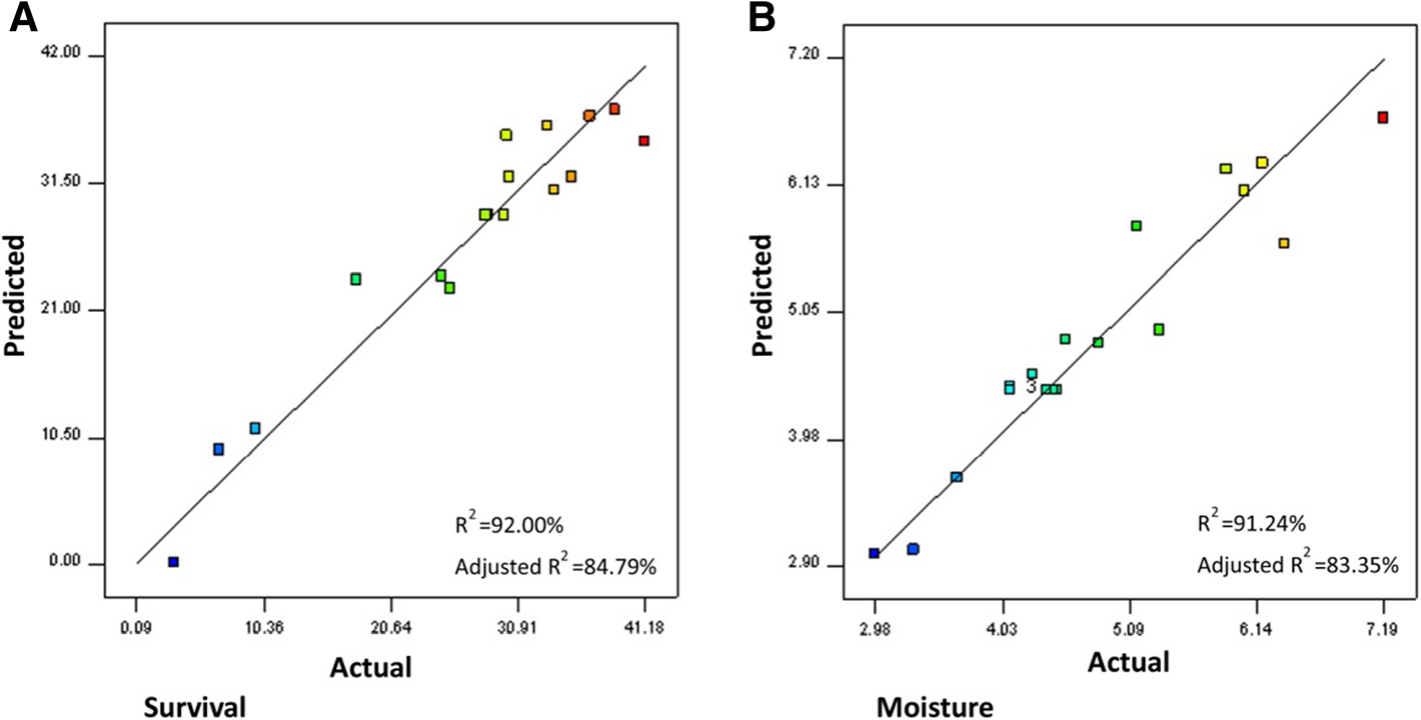
**Predicted vs. actual plot of: (A) survival rate and (B) moisture content of** ***B. bifidum*** **powder.**

Figure 1 represents predicted against actual values for survival and moisture content of *B. bifidum*, respectively. Actual values are the measured response data for a particular run, and the predicted values are evaluated using the approximating functions generated for the models (Eqs. (2)–(3)).

The fit quality of the second-order polynomial models equations (Eqs. (2)–(3)) were expressed by the coefficient of determination (*R*^2^). The value of *R*^2^ indicates that the quadratic equation is capable of representing the system under the given experimental domain. The coefficients of determination (*R*^2^) of the models were 0.92 for *Y*_1_ and 0.91 for *Y*_2_, which further indicates that the models (Eqs. (1)–(2)) were suitable for adequate representation of the real relationships among the variables. Since *R*^2^ and adjusted- *R*^2^ differ insignificantly, there is a good chance that the models include the important terms. Adequate precision is a measure of the range in predicted response relative to its associated error which provides a measure of the “signalto-noise ratio”. Its desired value is 4 or more [24]. In the present study, adequate precision was 13.24 for survival and 11.87 for moisture. Simultaneously, low values of the coefficient of variation (CV) (14.82 for survival and 9.16 for moisture) indicated good precision and reliability of the experiments. The CV as the ratio of the standard error of estimate to the mean-value of the observed response (as a percentage) was used as a measure of reproducibility of the model. All results showed that this model can be used to navigate the space defined by the CCD.

The p-value was used as a tool to check the significance of each coefficient. Low p-values indicate that the factor has a significant effect on results. A model term with a p-value < 0.05 is considered to be significant [28]. According to the p-values of the model terms (Table 3), A (Inlet air temperature), B (air pressure), interaction variable AB (Inlet air temperature × air pressure) and quadratic variable *A*^2^ are significant terms in the Survival of *B. bifidum* model. Furthermore, the only significant factor in moisture content of *B. bifidum* model is A (Inlet air temperature).

**Figure 2:**
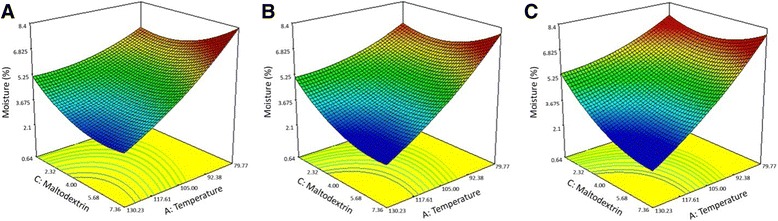
**The effect of temperature and maltodextrin concentration on the moisture content of** ***B. bifidum*** **powder.** Surface plot of the empirical model for moisture content (%) of *B. bifidum* powder at air pressures of **(A)** 4, **(B)** 5 and **(C)** 6 bars.

A negative sign for the coefficients of factors in the fitted models for *Y*_1_ and *Y*_2_ (Eq. 2 and 3) indicated that the level of the Survival of *B. bifidum* and the moisture content of *B. bifidum* increased with decreasing levels of factors. Also, the greatest coefficients of factor A (Inlet air temperature) revealed the high sensitivities of the both responses to this factor. Additionally the survival rate of *B. bifidum* was inversely proportional to air pressure and maltodextrin conc., but it seems that air pressure was more effective. Analysis of these models (Eq. 3) also showed that low moisture content is due to high maltodextrin conc. or application of high temperature or pressure, although the effect of temperature is significantly higher than other factors. To achieve a proper comprehension of the results, the predicted models are presented in Figure 2. The use of two-dimensional contour plots and three-dimensional surface plots of the regression model was highly recommended to obtain a graphical interpretation of the interactions [22,29].

Figure 2 depicts a three dimensional surface plot of the empirical model for moisture (%) as a function of three factors. Maltodextrin conc. and temperature were used for the RSM plots of moisture (%), while air pressure was increased from 4 bar to 5 bar and then 6 bar from left to right. As shown in Figure 2, at all air pressures, the lowest moisture was achieved at the highest concentrations of maltodextrin (7.36) and temperature (130.23). The results imply the need for application of more maltodextrin for having minimum moisture at the highest temperature. According to the surface plots, at the lowest maltodextrin conc. (0.64) and temperature (79.77) the moisture (%) increased by decreasing the air pressure from left to right. The moisture decreased when at the highest temperature (130.23), maltodextrin conc. increased, and vice versa. It was also true while at the highest conc. of maltodextrin (7.36), the temperature increased to its highest level.

However, at the lowest temperature (79.77), specifically at air pressure ≥5, decreasing the maltodextrin to 4%, resulted in lower moisture content, which may have been due to the more inhibitory effect of the maltodextrin concentration at air pressure ≥5. These results indicate that the measure of maltodextrin was critical for moisture of powder, which depends on inlet air temperature and air pressure.

**Figure 3:**
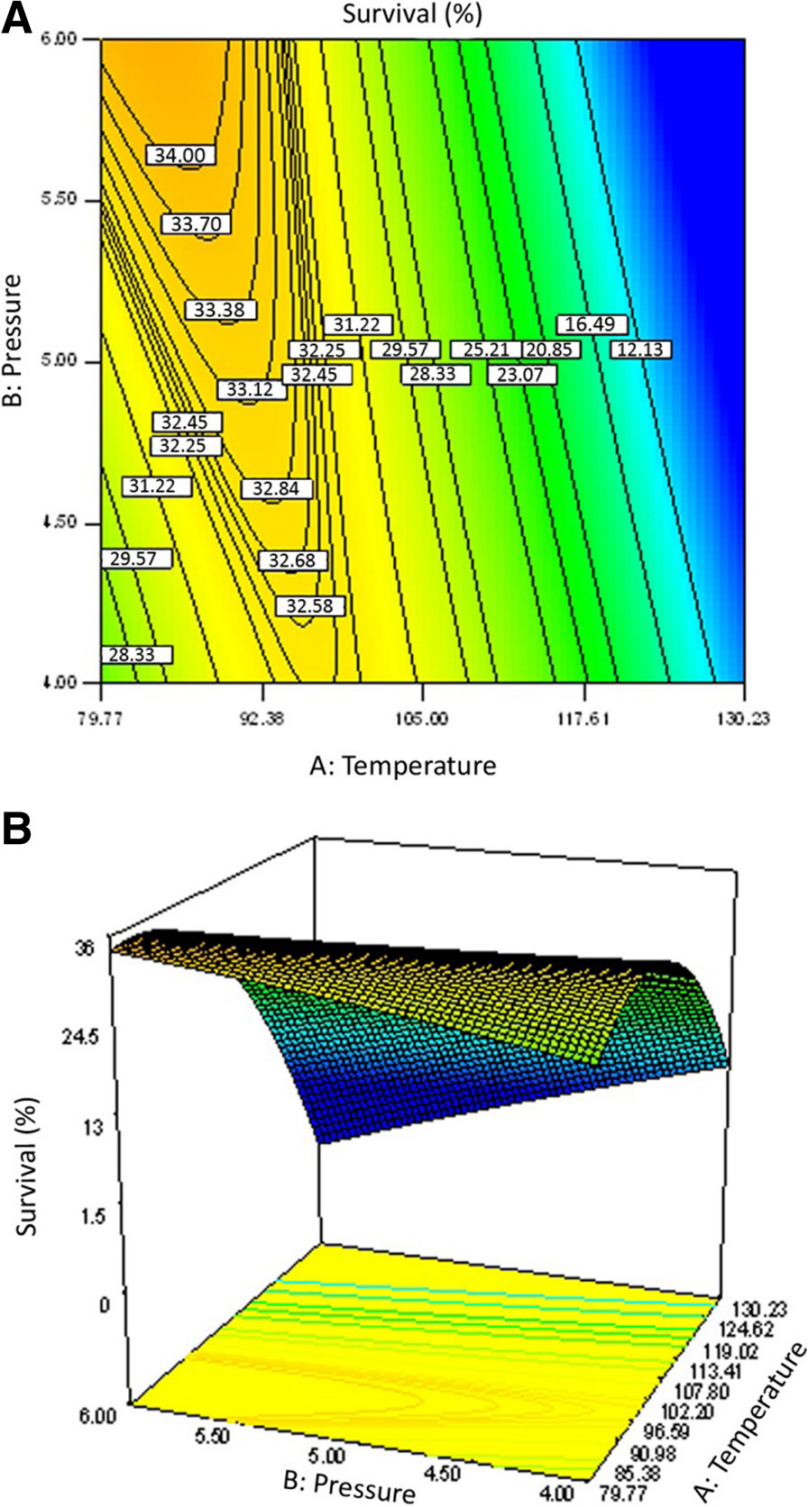
**The effect of temperature and air pressure on survival of** ***B. bifidum*** **. (A)** Contour and **(B)** 3D plots of *B. bifidum* survival at different temperatures and air pressure during spray drying. Maltodextrin concentration was kept at fixed 4%.

The dependence of the survival of *B. bifidum* on temperature and air pressure at 4% maltodextrin is depicted in Figure 3. The survival rate of *B. bifidum* increased linearly as pressure was increased from 4 to 6 at temperature ≤105°C. At temperature >105°C, survival of *B. bifidum* increased linearly as pressure decreased from 6 to 4 bar. Therefore the effect of pressure on survival of *B. bifidum* depends on the operational temperature. A curvature type relationship existed between the survival of *B. bifidum* and the temperature at the lowest pressure (4 bar), survival of *B. bifidum* increased by increasing the temperature toward 105°C. Furthermore increasing the temperature resulted in lower bacterial survival rate. As shown in Figure 3, the highest survival rate of *B. bifidum* was achieved at high pressure (6 bar) and low temperature (79.77).

**Figure 4:**
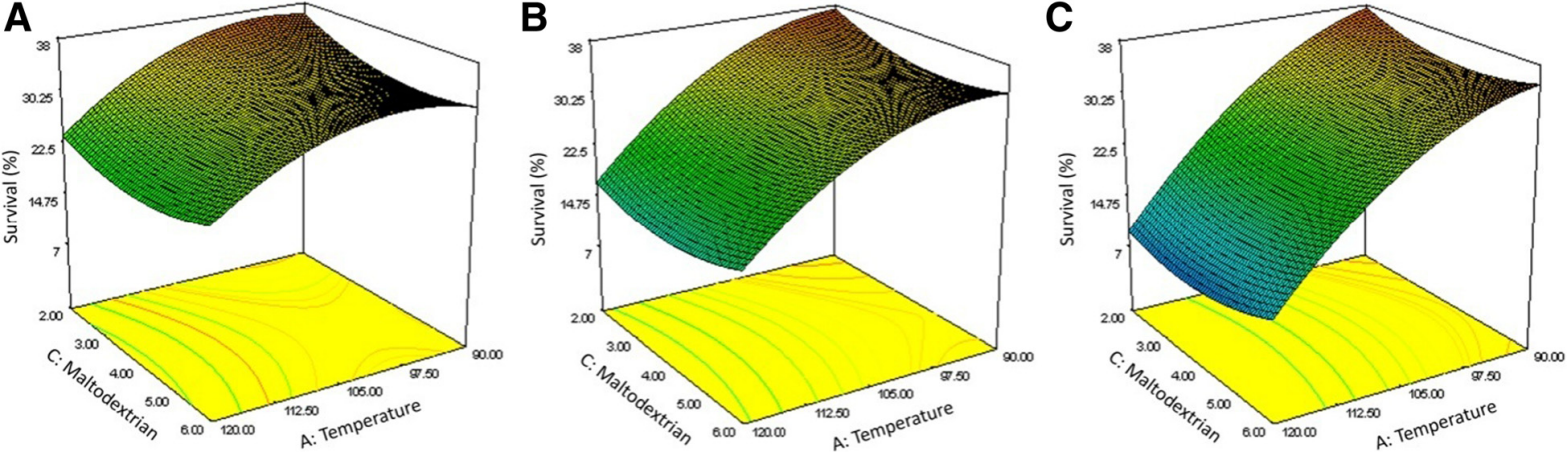
**Effect of temperature and maltodextrin concentration on the survival of** ***B. bifidum*** **.** Surface plot of the empirical model for survival (%) of *B. bifidum* at air pressures of **(A)** 4, **(B)** 5 and **(C)** 6 bars.

Figure 4 shows the effect of temperature and maltodextrin concentration on the survival of *B. bifidum*. The air pressure ranged from 4 bar to 6 bar from left to right. At the lowest temperature, particularly at air pressure ≥5, the survival rate was decreased by increasing the maltodextrin concentration to 5%. These results suggests maltodextrin content could highly affect survival of *B. bifidum* and low maltodextrin content could result to higher humidity of probiotic powder. Hence, maltodextrin concentration higher than 5% is highly recommended.

### Optimization

A simultaneous optimization technique was used for optimization of multiple responses by RSM. The objective of response surface optimization is to find a desirable location in the design space. Various optimum conditions can be considered, but the main goal of current experiment was to achieve maximal bacterial survival rate and keeping the moisture at as low as possible. According to numerical optimization by Design-Expert 7.0.0, the optimum was obtained by using the following spray drying conditions: inlet air temperature of 111.15°C, air pressure of 4.5 bar and maltodextrin conc. of 6.0%. Under these conditions, the survival of *B. bifidum* was 28.38% while the moisture content of the powder remained at 4.05%. These values are all in agreement with the results obtained from the three-dimensional surface plots.

Table 4 presents the results confirmation test and shows that verification experiments and predicted values from fitted correlations were in close agreement at a 95% confidence interval. These results confirmed the validity of the models.

**Table 4:** **Optimum process and validation experiment results at 95% confidence interval**

**Responses**	**Target**	**Predicted results**	**Confirmation test results**	**95% CI low**	**95% CI high**
Survival (%)	Maximize	28.38	29.78	23.95	32.83
Moisture (%)	Minimize	4.05	4.26	3.56	4.54

### The role of other culture media substances during spray draying

The main goal of current study was to achieve a high bacterial survival rate using cost effective media suitable for industrial scale production of probiotic powder. Both sucrose and glucose showed similar effect on bacterial growth but glucose did not have the protective effect of sucrose during spray drying. Previous studies used RSM as the carbon source for bacterial growth and also key protective substance in spray drying of bacteria. In current study RSM was replaced by inexpensive permeate. It owns all beneficial features of RSM and also contains vitamins like thiamine, riboflavin and niacin which are required for the growth of *B. bifidum*. Permeate was found to be the ideal medium for spray drying due to its protective proteins which prevent bacterial damage by stabilizing cell membrane components [30]. In addition, its calcium may form a protective layer. The solid ratio index was 20% for permeate, maltodextrin, sucrose and yeast extract had the best effect in bacterial count which is consistent with those reported by previous studies [31].

Different types of probiotic adherent fibers such as fructo-oligosaccharide (FOS) and galacto-oligosaccharide (GOS) are usually used as a carrier in the culture medium of bifidobacteria and lactobacilli during spray and freeze drying. Maltodextrin was used in the culture medium as the adhesion agent. Despite the structural and functional similarities of maltodextrinwith dextrose, maltodextrin protects bacteria much better than Polydextrose at the high temperature and pressure and has the advantage of cost-effectiveness compared to inulin [32]. It has also been considered as prebiotic which stimulates probiotic growth.

### Role of spray dryer factors

The results showed that air temperature had the main effect on residual moisture of bacterial products, as well as bacterial survival rate. Since bacteria are exposed to outlet temperature in different parts of spray dryer, it should not be above 75°C which causes serious damage to susceptible bacteria during spray drying process. Also it should not be too low (below 60°C) which could end up with high moisture content (up 7%). Protective effects of polysaccharides are due to the ability of the sugars to form a high viscous glassy matrix during dehydration. Moisture uptake would decrease the glass transition temperatures of the system, and consequently a transition of the glass state of sugar towards the rubbery state (denitrification) could occur which might decrease the stability of spray dried powder. Therefore, the best moisture content of 4-7%, was achieved in the outlet temperature of 60-80°C.

## Conclusions

Statistical modeling and optimization of spray drying of *Bifidobacterium bifidum* PTCC 1644 was investigated. The thermal compliances of an acid-bile-adopted probiotic strain was increased to 75°C using induced environmental stress condition. Permeate and maltodextrin were used as the protecting agents instead of reconstituted skim milk reported by other researchers. The RSM-CCD was used for statistical analysis and optimization of the process. The effect of inlet air temperature, air pressure and maltodextrin concentrations on survival and moisture of spray dried *B. bifidum* were assessed. Two quadratic models for the responses were developed. Temperature had the most significant effect on spray drying of *B.bifidum*. Maximum survival rate of 28.38% and minimum moisture content of 4.05% was achieved at T = 111.15°C, P = 4.5 bar and maltodextrin content of 6%.

Powders of live beneficial probiotic bacterial cultures could be achieved by preadaptation of the individual strains to gastrointestinal as well as other environmental factors and further addition of selected protective polysaccharides into the culture media before spray drying.
